# Noninvasive 7 tesla MRI of fatal craniocerebral gunshots – a glance into the future of radiologic wound ballistics

**DOI:** 10.1007/s12024-020-00300-w

**Published:** 2020-09-12

**Authors:** Dominic Gascho, Eva Deininger-Czermak, Niklaus Zoelch, Carlo Tappero, Stefan Sommer, Natalie Hinterholzer, Michael J Thali

**Affiliations:** 1 Department of Forensic Medicine and Imaging, Zurich Institute of Forensic Medicine, University of Zurich, Winterthurerstrasse 190/52, CH-8057 Zurich, Switzerland; 2 Institute of Diagnostic and Interventional Radiology, University Hospital Zurich, Zurich, Switzerland; 3 Department of Psychiatry, Psychotherapy and Psychosomatics, Hospital of Psychiatry, University of Zurich, Zurich, Switzerland; 4 Department of Radiology, Hôpital Fribourgeois, Fribourg, Switzerland; 5 Siemens Healthcare AG, Zurich, Switzerland; 6 SCMI, Swiss Center for Musculoskeletal Imaging, Balgrist Campus AG, Zurich, Switzerland

**Keywords:** 7 T MRI, Siemens MAGNETOM Terra, Magnetic resonance imaging, Gunshot wound, Radiologic wound ballistics, Forensic radiology, Virtual autopsy, Virtopsy

## Abstract

Compared to computed tomography (CT), magnetic resonance imaging (MRI) provides superior visualization of the soft tissue. Recently, the first 7 Tesla (7 T) MRI scanner was approved for clinical use, which will facilitate access to these ultra-high-field MRI scanners for noninvasive examinations and scientific studies on decedents. 7 T MRI has the potential to provide a higher signal-to-noise ratio (SNR), a characteristic that can be directly exploited to improve image quality and invest in attempts to increase resolution. Therefore, evaluating the diagnostic potential of 7 T MRI for forensic purposes, such as assessments of fatal gunshot wounds, was deemed essential. In this article, we present radiologic findings obtained for craniocerebral gunshot wounds in three decedents. The decedents were submitted to MRI examinations using a 7 T MRI scanner that has been approved for clinical use and a clinical 3 T MRI scanner for comparison. We focused on detecting tiny injuries beyond the wound tract caused by temporary cavitation, such as microbleeds. Additionally, 7 T T_2_-weighted MRI highlighted a dark (hypo intense) zone beyond the permanent wound tract, which was attributed to increased amounts of paramagnetic blood components in damaged tissue. Microbleeds were also detected adjacent to the wound tract in the white matter on 7 T MRI. Based on the findings of radiologic assessments, the advantages and disadvantages of postmortem 7 T MRI compared to 3 T MRI are discussed with regard to investigations of craniocerebral gunshot wounds as well as the potential role of 7 T MRI in the future of forensic science.

## Introduction

In 2017, the first magnetic resonance imaging (MRI) scanner operating at the very high magnetic field strength of 7 Tesla (T) received a CE mark and obtained FDA clearance for clinical use. With respect to continuation of the virtual autopsy (virtopsy) approach [1, 2], evaluating the diagnostic power of this 7 T MRI unit for forensic interests, such as diagnostic assessments of gunshot wounds in radiologic wound ballistics, was deemed essential.

In craniocerebral gunshots, the energy transferred by the penetration of the projectile into the cranium can cause parenchymal injuries to tissue beyond the permanent cavity (wound tract) along the bullet’s trajectory. In the so-called temporary cavity beyond the wound tract, shearing, compressing, and stretching forces exerted during outward acceleration from the wound tract can cause brain tissue damage [3–8], which is referred to as cavitation injury [9]. In the context of MRI, parts of the temporary cavity can present a different signal intensity than the surrounding healthy tissue, which was described as having the appearance of “enlightened” or “translucent” zones around the missile track [10, 11]. To date, the diagnostic potential of noninvasive postmortem MRI in craniocerebral gunshots has only been investigated at field strengths of 1.5 T [1, 10] or 3 T [12]. A higher field strength, particularly 7 T MRI, has the potential to provide a higher signal-to-noise ratio (SNR) due to an increase in spin polarization and a higher resonance frequency. The increased SNR can be directly exploited to improve image quality and invested in attempts to increase the resolution (or shorten the scan times) of images obtained using 7 T MRI. Additionally, the maximum achievable contrast-to-noise ratio (CNR) increases as the SNR and field strength increase [13]. Therefore, ultra-high-field 7 T MRI may be beneficial for the detection of small injuries. Susceptibility-weighted imaging (SWI) highlights signal dropouts in the vicinity of structures with largely different susceptibilities than those generally found in the surrounding tissue, such as venous blood, hemosiderin, and methemoglobin in hemorrhages [13]. Since the differences in magnetic fields among brain regions with different tissue magnetic susceptibilities increase linearly with increasing field strength, spin dephasing occurs faster at 7 T than at lower field strengths [14]. Considering the enhanced susceptibility effect and the high spatial resolution obtained at 7 T, microhemorrhages, also referred as microbleeds [15], may be easier to detect and amenable to more detailed analysis when using 7 T MRI. Unfortunately, compared to lower field strengths, these ultra-high field strengths also have drawbacks. Among these drawbacks is the finding that an increased B_0_ and an increased B_1_ inhomogeneity may impede improvement of the radiologic assessment and detection of craniocerebral gunshot injuries when using 7 T.

This article evaluates the application of the first 7 T MRI scanner ever approved for clinical use with regard to potential diagnostic benefits for the detection of craniocerebral gunshot injuries examined postmortem and in situ.

## Materials and methods

The study population consisted of three forensic cases of gunshot wounds and one control case with edema but without cerebral injuries. Thorough external examinations were carried out by forensic pathologists at the locations where the bodies were found before the decedents were submitted to radiologic imaging as part of forensic judicial investigations.

### Case histories

Case 1 (a 41-year-old male) was found in a forest at an approximate ambient temperature of 10 °C/50 °F during the day and 0 °C/32 °F during the night. During the external examination performed at the place where the body was found, the estimated time since death was 1–3 weeks. A Glock 17 Gen 4 pistol (caliber: 9 mm) was found next to the decomposed cadaver.

Case 2 (a 77-year-old male) was found in a public park. When the body was found, the ambient temperature was approximately 5 °C/41 °F, and the estimated time since death was 9 to 12 h. An SIG Sauer P226 pistol (caliber: 9 mm) was found next to the decedent.

Case 3 (a 64-year-old male) was found in a public area. As the decedent had informed the police of his location prior to his death, he was found immediately after a suicidal gunshot. An SIG Sauer P210 pistol (caliber: 9 mm) was found next to the body.

The control case (a 39-year-old female) was found dead in her flat. The decedent did not present injuries on external examination. The prosecutor commissioned radiologic examinations for further clarification of the cause of death.

### Postmortem imaging

First, computed tomography (CT) scans were performed using a 128-slice CT scanner (*SOMATOM Definition Flash, Siemens Healthcare, Erlangen, Germany*). In addition to a whole body scan [16], a high-resolution CT scan of the head was also performed (tube voltage: 120 kVp; tube current: 360 mAs; slice thickness: 0.4 mm). The CT scan was performed to exclude the presence of potentially ferromagnetic bullet fragments and to detect bone damage, which allowed identification of the entrance and exit wounds.

The 7 T MRI data were acquired using an actively shielded system recently approved for clinical use (*MAGNETOM Terra, Siemens Healthcare, Erlangen, Germany*). The 7 T MRI protocol included the following sequences:

- T_2_-SPACE (T_2−_weighted, TR/TE [ms]: 4000/118; encoded voxel dimensions [mm]: 0.71 × 0.71 × 0.67; orientation: sagittal; scan time: 7:34 min),
- MP2RAGE (T_1−_weighted, TR/TE [ms]: 4500/2; encoded voxel dimensions [mm]: 0.65 × 0.65 × 0.63; orientation: sagittal; scan time: 10:45 min),
- SWI (susceptibility-weighted, TR/TE [ms]: 21/14; encoded voxel dimensions [mm]: 0.2 × 0.2 × 1.5; orientation: transversal; scan time: 10:40 min), and
- Spoiled 3D gradient echo (GRE) (T_1_-weighted, TR/TE [ms]: 4.2/1.5; encoded voxel dimensions [mm]: 04 × 0.4 × 0.5; orientation: sagittal; scan time: 6:15 min).

For comparison, 3 T MRI examinations were performed using a clinical MRI scanner (*Achieva 3.0 TX, Philips Healthcare, Best, the Netherlands*) immediately before or after the 7 T MRI examination. The 3 T MRI protocol included the following sequences:

- T_2_-TSE-SPAIR (T_2_-weighted, TR/TE [ms]: 1500/230; encoded voxel dimensions [mm]: 0.8 × 0.8 × 1.6; orientation: transversal; scan time: 6:00 min),
- T_1_-TFE (T_1_-weighted, TR/TE [ms]: 9.4/4.6; encoded voxel dimensions [mm]: 1.0 × 1.0 × 2.0; orientation: sagittal; scan time: 8:50 min), and
- VenBold (susceptibility-weighted, TR/TE (shifted [10]) [ms]: 18/26; encoded voxel dimensions [mm]: 0.8 × 0.8 × 1.6; orientation: transversal; scan time: 6:21 min).

The interval between the 7 T and 3 T MRI scans was approximately one hour. The rectal temperatures of the decedents were 9–11 °C (control case: 12 °C) when measured after the MRI examinations.

### Radiologic assessment

The radiologic assessment was performed in collaboration with two radiologists with experience in forensic medicine. The MRI examinations were assessed with regard to (a) detection of residues, (b) identification of the entrance and exit wounds, (c) indications of the shot direction based on soft tissue injuries, (d) visualization of the wound tract, and (e) identification of the injuries beyond the wound tract based on a 5-point Likert scale (1 = not visible or not assessable, 2 = poor, 3 = average, 4 = good, 5 = very good). The external examination and CT served as the gold standard for a and b. No gold standard was available for c, d, and e. The grading of the T_1_-weighted, the T_2_-weighted, and the susceptibility-weighted images from 7 T MRI were compared with those from the 3 T MRI examinations.

**Fig. 1:**
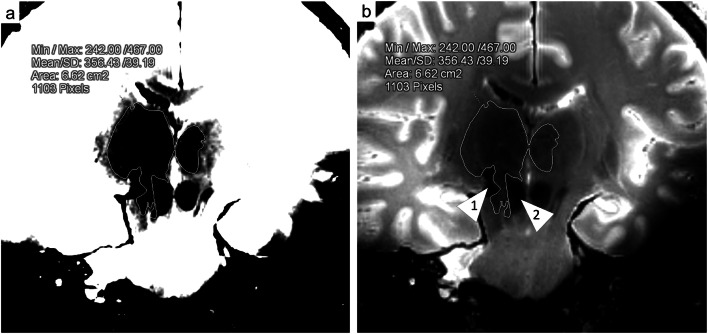
Coronal view of the 7 T T_2_-weighted images aligned to the wound tract in case 1. The area of the zone with low MRI signal intensity was measured by drawing freehand ROIs on every slice that delineated such a dark zone (**a**: window: 100, center: 450; **b**: window: 550, center: 550). The wound tract (arrowhead 1) and anatomical structures with low MRI signal intensities (arrowhead 2: nucleus ruber) were excluded from the ROIs

Increased amounts of hemosiderin and methemoglobin in damaged tissue adjacent to the permanent wound tract were assumed to cause signal dropouts or rather dark (hypo intense) zones. If visible, the sizes of such dark zones were calculated by drawing freehand regions of interest (ROIs) covering the zone of low MRI signal intensity beyond the wound tract. ROIs were drawn on each slice that delineated such a dark zone in the cross section to the wound tract (Fig. 1). For this purpose, the T_2_-weighted datasets were aligned to the wound tract.

### Statistical analysis

The Wilcoxon test was used to reveal statistically significant differences between Likert scale grades and between the ROI-defined areas (significance level: *p* < 0.05). The statistical analyses were performed using the Statistical Package for the Social Sciences (SPSS, International Business Machines Corporation (IBM), Armonk, NY, USA).

## Results

The three non-control cases presented perforating gunshot wounds. The manners of death were unnatural (suicide), and the causes of death were determined to be central respiratory paralysis due to perforating craniocerebral gunshot wounds. The cause of death in the control case with edema but without cerebral injuries was determined to be intoxication by morphine overdose according to toxicological analysis.

As expected, the decedents with craniocerebral gunshot wounds presented severe brain tissue damage on CT consistent with close-range shots using 9 mm ammunition. Gunshot residues were detected at the entrance wound on CT in case 2. Metallic fragments from the bullets were not visible on the CT scans. The missile track was determined to be frontooccipital in case 1, temporoparietal in case 2, and palatoparietal in case 3 (with a submental entrance wound) based on bone damage observed on CT. This damage included inwardly and/or outwardly beveled fractures. Due to severe soft tissue damage, both the radiologic assessment of the brain tissue and the identification of injured intracerebral structures were severely impeded on CT.

**Fig. 2:**
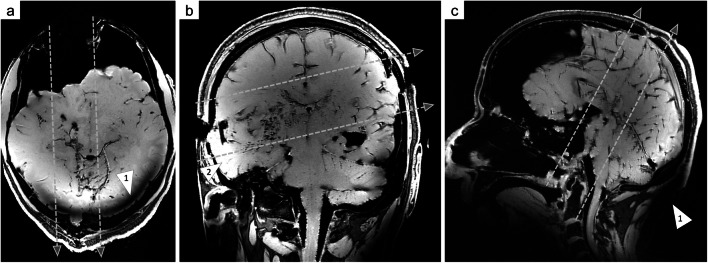
Multiplanar reformats of a spoiled 3D GRE sequence aligned to the individual bullet path (bullet trajectory: dashed arrows). This 7 T MRI sequence provided a good compromise between visualization of bone damage and soft tissue injuries. Case 1 (**a**: transversal view) demonstrated a transverse trajectory (entrance wound: frontal bone; exit wound: occipital bone), case 2 (**b**: coronal view) presented a horizontal trajectory (entrance wound: right temporal bone; exit wound: left parietal bone), and case 3 demonstrated a vertical trajectory (entrance wound: submental soft tissue; exit wound: parietal bone). B_1_ inhomogeneity was visible as brightened structures far from the image center (a: arrowhead 1). Signal dropouts (c: arrowhead 1) were observed in the lower occipital region because the field of view was smaller due to the absence of a body coil and B_1_ inhomogeneity

A spoiled 3D GRE sequence delineated the missile tracks by depicting bone damage and indicated that the wound tracts ran through the brain tissue (Fig. 2).

**Fig. 3:**
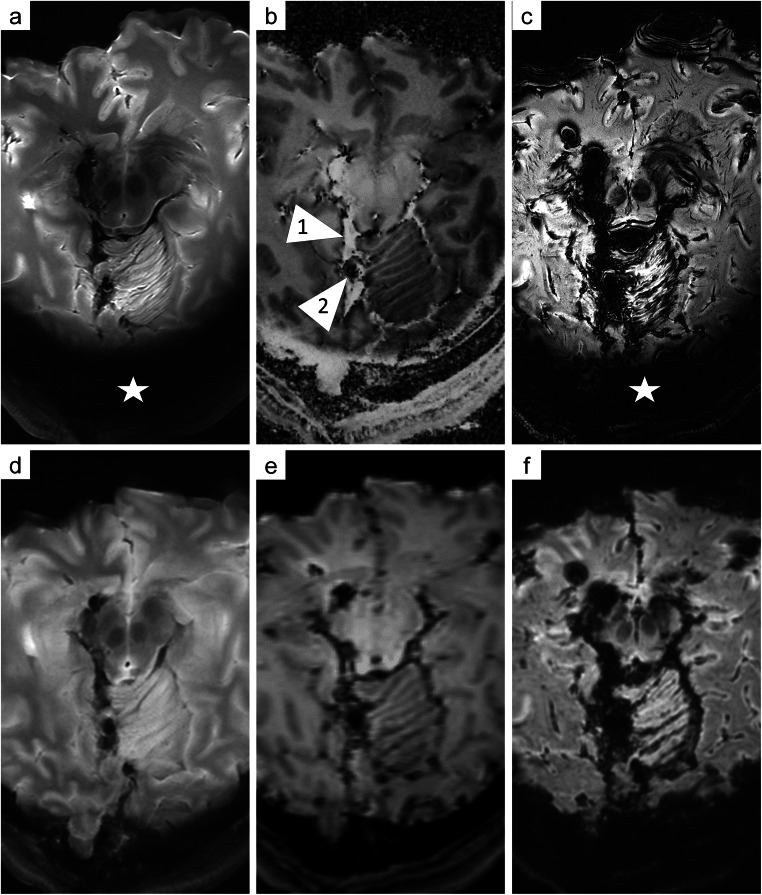
Comparison of 7 T images (**a**: T_2_-SPACE; **b**: MP2RAGE; **c**: SWI) and 3 T images (**d**: T_2_-TSE-SPAIR; **e**: T_1_-TFE; **f**: VenBold) obtained in case 1 and aligned to the bullet path. The wound tract and soft tissue injuries along the bullet path, such as the injury to the pedunculus cerebri, were delineated with high resolution on 7 T MRI but were blurry on 3 T images. The T_2_-SPACE sequence (**a**) demonstrated higher spatial resolution and soft tissue contrast, which allowed a detailed depiction of the wound tract to be obtained. The MP2RAGE sequence (**b**) allowed clear distinction between blood (arrowhead 1: hyper intense, bright) and gas (arrowhead 2: hypo intense, dark) and additionally provided high contrast between gray and white matter. Although the SWI sequence (**c**) presented very high spatial resolution, the radiologic assessment was severely impeded by intracranial gas cavities. Signal dropout was observed in the lower occipital region (asterisk)

**Fig. 4:**
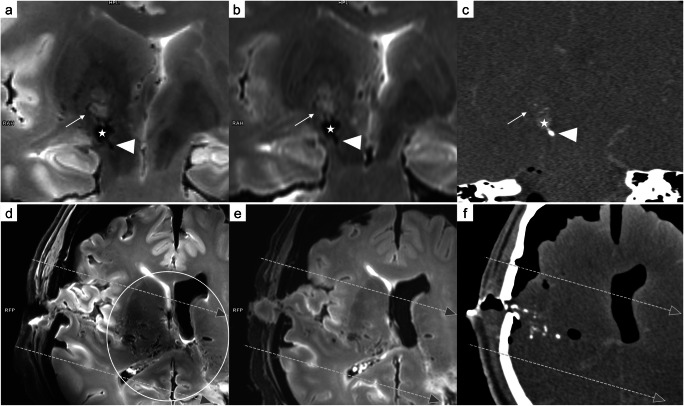
7 T T_2_-weighted images (**a** and **d**), 3 T T_2_-weighted images (**b** and **e**), and CT reconstruction (**c** and **f**). In a cross-section of the bullet path in case 1 (a-c: coronal view), soft tissue damage (arrow) was detected beyond the wound tract (asterisk). Differentiation between blood and bone fragments (arrowhead) was barely possible based on only the T_2_-weighted sequences. Furthermore, the 7 T images delineated numerous cavitation injuries in the region of the thalamus (circle) along the trajectory (dashed arrows)

**Fig. 5:**
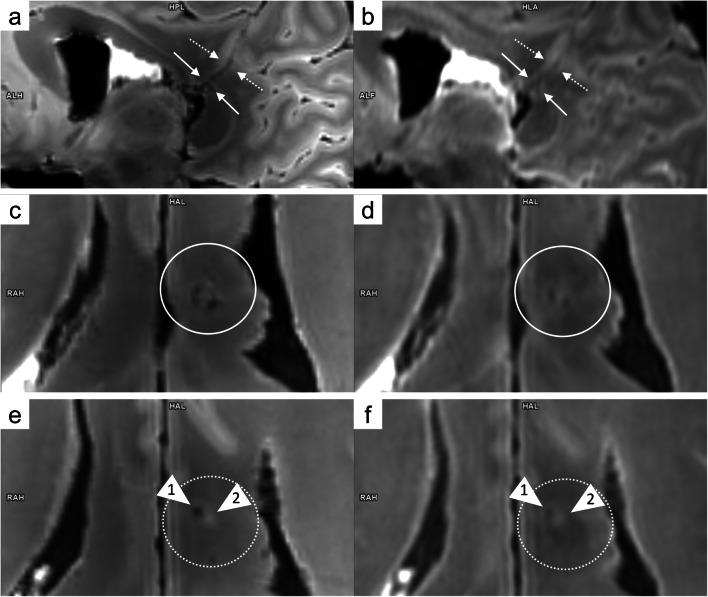
Comparison of 7 T T_2_-weighted images (**a**: sagittal view; **c** and **e**: transversal view) and 3 T T_2_-weighted images (**b**: sagittal view; **d** and **f**: transversal view) in case 3. The wound tract is identifiable on 7 T MRI (a: arrows) but difficult to delineate on 3 T MRI (**b**: arrows). In a cross-section of the bullet path (**c** and **d**: aligned according to the white arrows in **a** and **b**; **e** and **f**: aligned according to the white dashed arrow in **a** and **b**), 7 T allowed visualization of tiny injuries along the bullet path (**c**: white circle, hyper intense/dark structures) that were barely identifiable on 3 T (**d**: white circle). Furthermore, the tiny wound tract through the white matter was visible only on 7 T MRI (**e**: arrowhead 1). A small bright/hyper intense region was identified directly adjacent to the wound tract (arrowhead 2)

**Fig. 6:**
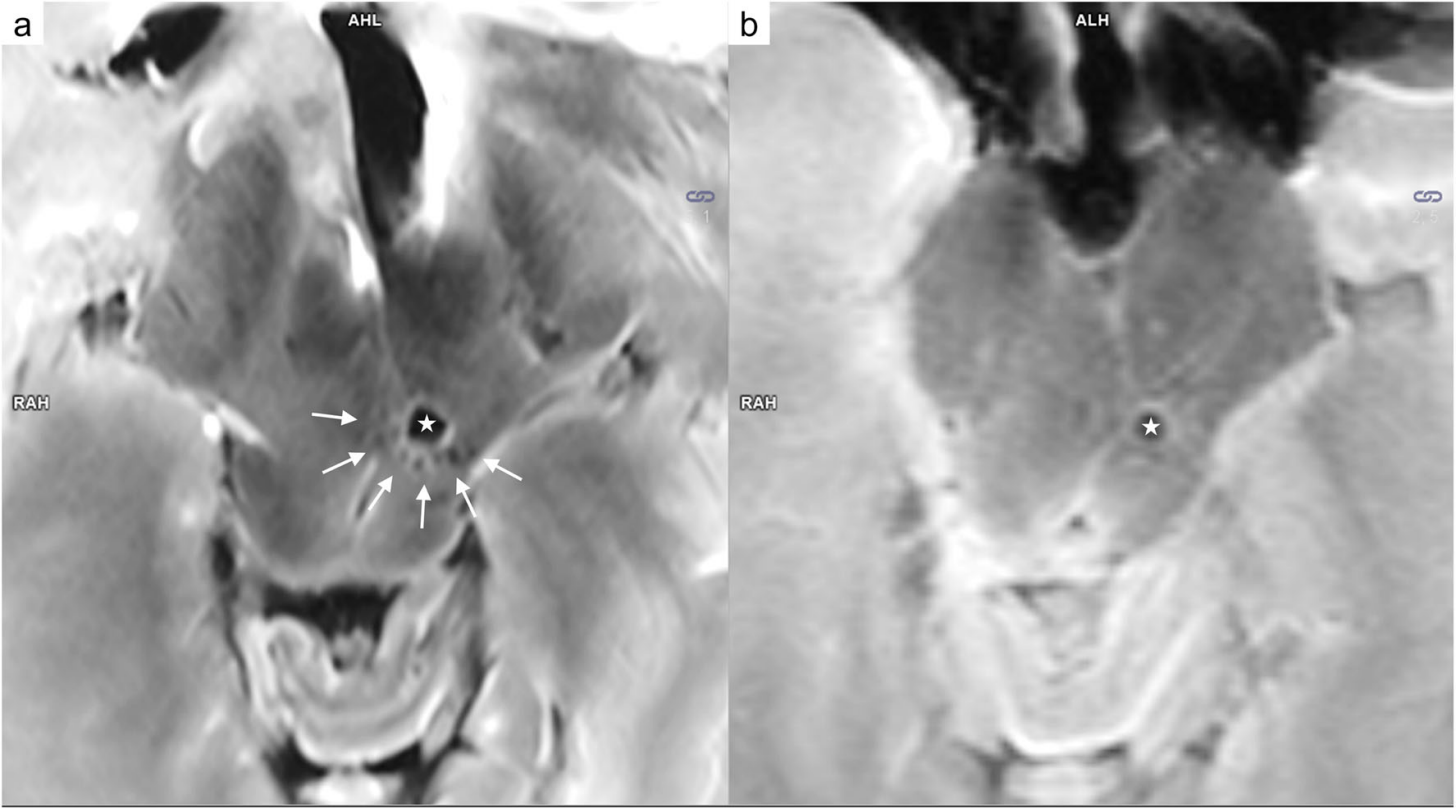
Comparison of 7 T T_2_-SPACE images (**a**) and 3 T T_2_-TSE-SPAIR images (**b**) obtained in case 3 in a transversal view. Only 7 T MRI enabled identification of several microbleeds (arrows: tiny dark/hypo intense dots) surrounding the wound tract (asterisks)

The 7 T T_2_ images presented higher contrast between the different structures of the brain tissue than the 3 T T_2_ images (Fig. 3a/d). The 7 T T_2_-SPACE sequence enabled a precise depiction of soft tissue injuries beyond the wound tract and a detailed depiction of anatomical structures. Cavitation injuries were detected beyond the wound tract, although identification of bone fragments and gas was challenging on MRI (Fig. 4). The wound tract through the collapsed brain tissue was more difficult to identify on 3 T than on 7 T (Fig. 5). Several microbleeds were visible only on 7 T MRI (Fig. 6). The Likert scale grades of T_2_-weighted images (Table 1) were significantly higher (*p* = 0.030) on 7 T MRI than on 3 T MRI.

**Table 1:** Likert scale grading: mean (± standard deviation)

	3 T MRI	7 T MRI
T_2_-weighted		
residues (shot distance)	1.0 (± 0.0)	1.0 (± 0.0)
entrance and exit wounds (shot direction)	2.7 (± 0.9)	3.0 (± 1.4)
soft tissue injuries indicating the shot direction	1.0 (± 0.0)	1.0 (± 0.0)
wound tract	3.0 (± 0.8)	4.0 (± 0.8)
injuries beyond the wound tract	2.3 (± 0.5)	4.0 (± 0.0)
T_1_-weighted		
residues (shot distance)	1.0 (± 0.0)	1.0 (± 0.0)
entrance and exit wounds (shot direction)	2.3 (± 0.5)	2.0 (± 0.0)
soft tissue injuries indicating the shot direction	1.0 (± 0.0)	1.0 (± 0.0)
wound tract	2.3 (± 0.5)	2.7 (± 0.5)
injuries beyond the wound tract	1.7 (± 0.5)	2.3 (± 0.5)
susceptibility-weighted		
residues (shot distance)	1.0 (± 0.0)	1.0 (± 0.0)
entrance and exit wounds (shot direction)	1.0 (± 0.0)	1.0 (± 0.0)
soft tissue injuries indicating the shot direction	1.0 (± 0.0)	1.0 (± 0.0)
wound tract	1.7 (± 0.5)	1.7 (± 0.5)
injuries beyond the wound tract	1.0 (± 0.0)	1.0 (± 0.0)

**Fig. 7:**
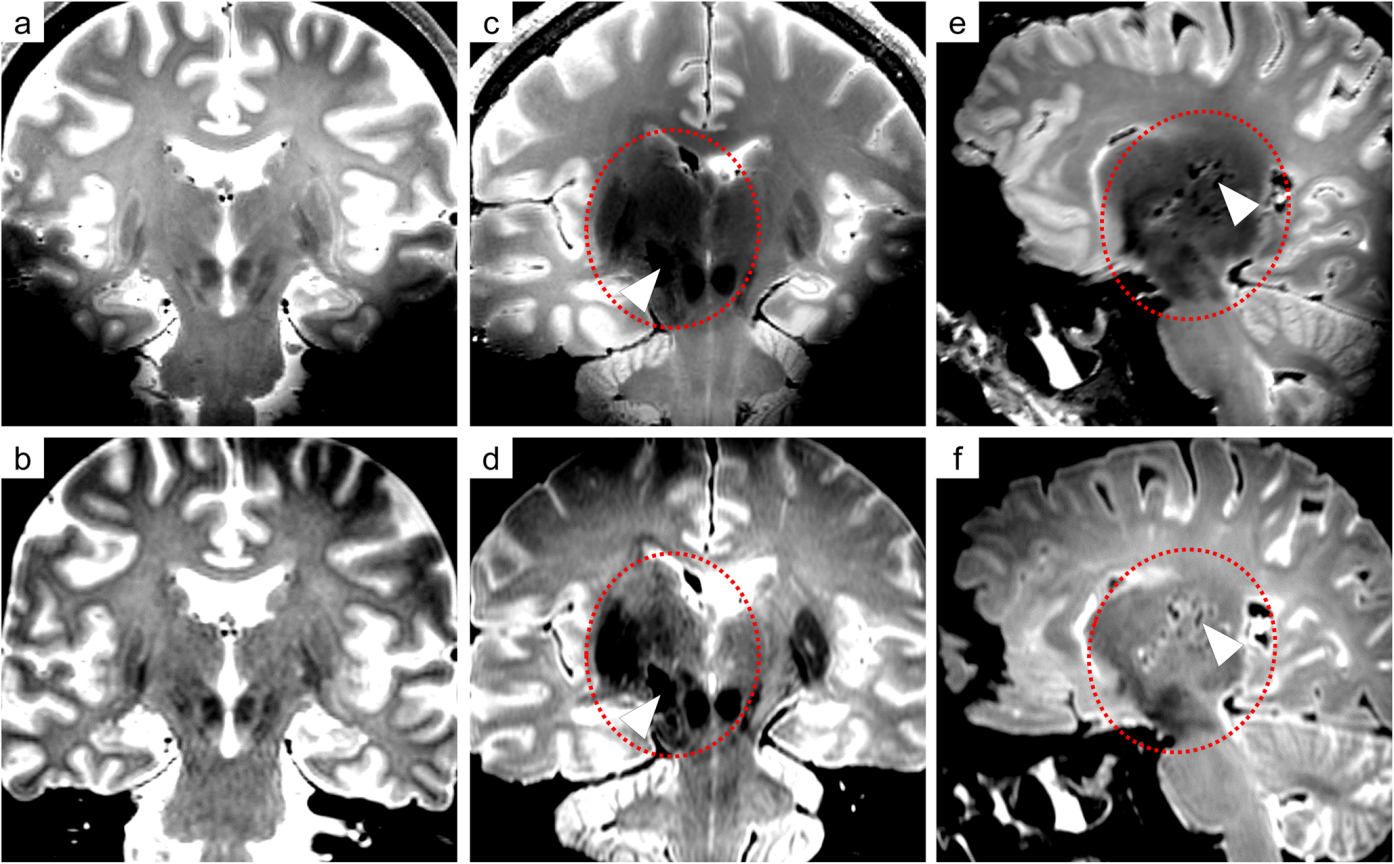
Comparison of the control case (**a** and **b**) with case 1 (**c** and **d**) and case 2 (**e** and **f**) in terms of the extent of cavitation injuries delineated by a dark/hypo intense zone (red circle) beyond the wound tract (arrowhead) on 7 T MRI (upper row) and on 3 T MRI (lower row). The dark/hypo intense zone was defined as damaged tissue. On 7 T MRI (c and e), the damaged tissue beyond the wound tract could be highlighted

Furthermore, in all three cases with craniocerebral gunshot injuries, a dark (hypo intense) zone could be delineated adjacent to the wound tract in the region of the diencephalon and mesencephalon on 7 T MRI, which was hardly detectable on 3 T MRI and was not observed in the control case (Fig. 7). In all three cases, the dark zones were significantly larger on 7 T MRI than on 3 T MRI according to ROI measurements (case 1: *p* = 0.001; case 2: *p* = 0.002; case 3: *p* = 0.030).

Compared to the high-resolution 7 T T_1_ images, the 3 T T_1_ images were blurrier (Fig. 3b/e). The MP2RAGE sequence allowed clear distinction between blood (bright/hyper intense) and gas (dark/hypo intense) along the wound tract, which was barely feasible on 3 T T_1_ images. Furthermore, the MP2RAGE sequence presented high contrast between gray and white matter. However, the Likert scale grades of the T_1_-weighted images (Table 1) were not significantly different (*p* = 0.317) between 7 T MRI and 3 T MRI.

The susceptibility-weighted images demonstrated a clear difference with regard to spatial resolution between 7 T and 3 T MRI (Fig. 3c/f). However, the large number of gas cavities distributed inside the damaged soft tissue impeded the identification of small bleeds. The Likert grades of the susceptibility-weighted images (Table 1) were not significantly different (*p* = 1.000) between 7 T and 3 T MRI.

The prosecutor waived additional autopsies in all three cases, which were finally determined to be suicides by gunshots to the head. Therefore, comparisons with macroscopic or histologic images could not be conducted in this study.

## Discussion

The use of an ultra-high-field 7 T MRI scanner allows noninvasive acquisition of detailed information on gunshot injuries. We found that 7 T MRI allowed visualization of tiny soft tissue injuries beyond the wound tract, such as microbleeds, that were not visible on 3 T MRI (or CT). Furthermore, dark (hypo intense) zones beyond the permanent wound tract were clearly delineated on 7 T MRI, which might be an indication of the extent of injured tissue.

Craniocerebral gunshot wounds are classified as (mild) traumatic brain injuries [4, 6]. While the use of MRI to detect microbleeds in traumatic brain injuries has been described in the literature [17–19], the detection of microbleeds in craniocerebral gunshot wounds by MRI is presented for the first time in the current article. Susceptibility-weighted sequences have been applied to detect microbleeds in previous studies of traumatic brain injuries [17–19]; in contrast, in the present study, the T_2_-weighted sequence was preferred to identify microbleeds and cavitation injuries on 7 T MRI. The diagnostic value of the 7 T SWI sequence was reduced by the large amount of intracranial gas. Severe damage to the brain tissue impeded diagnostic assessments of the forensic cases in this study. Small-caliber gunshots from a greater distance may be more appropriate for the use of susceptibility-weighted sequences since less severe bone and soft tissue damage is expected in such cases. To detect blood within the wound tract or distinguish between blood and gas within the wound tract, susceptibility-weighted sequences did not provide any additional information in the present study. When using the MP2RAGE sequence, however, blood within the wound tract could be clearly distinguished from gas. MP2RAGE combines two images with different inversion times to minimize proton density, reception and transmit field inhomogeneity and T_2_* contributions to the T_1_-weighted image [20]. Compared to standard gradient echo sequences, this sequence displays arteries very brightly (hyper intense) [20], and this effect has been used to obtain angiograms without the application of contrast media [20, 21]. Concerning the detection of microbleeds, however, the MP2RAGE images did not produce highlighted small hemorrhages in the present cases of craniocerebral gunshot wounds. In addition to the distinction between blood and gas within the wound tract, the 7 T T_1_-weighted sequence also provides high contrast between gray and white matter compared to 3 T images [22], which may be of interest for other medico-legal investigations involving studies of morphometry [23] or tissue segmentation [24].

The dark (hyper intense) zones adjacent to the wound tract on the T_2_-weighted images were attributed to increased amounts of paramagnetic blood components in damaged tissue. These dark zones were only observed in the region of the diencephalon and mesencephalon and not in the white or grey matter, which is probably related to the different compositions of these tissues. The low MRI signal in the tissue adjacent to the wound tract was delineated on the T_2_-weighted images, while the susceptibility-weighted images were less appropriate in this study. Since 7 T MRI is more sensitive to this effect than 3 T MRI, the larger zones on 7 T T_2_-weighted images were assumed to indicate the damaged tissue more precisely than those on the 3 T T_2_-weighted images. Certainly, this assumption requires further investigation by comparing the extent of these dark zones with macroscopic and histologic examinations.

Compared to 3 T MRI, 7 T MRI also has several drawbacks, such as a smaller field of view due to the absence of a body coil and B_1_ inhomogeneity. Furthermore, the increase in magnetic field strength affects tissue relaxation times [25]. Thus, in addition to the influence of body temperature on postmortem neuroimaging [26], soft tissue contrast also differs between 3 T MRI and 7 T MRI when identical acquisition parameters are used. With regard to body temperature, rectal temperature did not noticeably increase during either 3 T or 7 T MRI examinations.

With regard to the images presented in this article, direct comparisons between images acquired at 7 T and at 3 T notably have only limited validity since MRI scanners produced by different manufacturers have different implementations for the radio-frequency system, the applied sequences and the reconstruction of the images. Therefore, additional calculations, such as those for SNRs or CNRs, were omitted from the comparison between 3 T and 7 T MRI.

Although several postmortem studies have demonstrated the diagnostic potential of MRI in forensic neuroimaging [27–31], medico-legal societies still do not consider MRI a substitute for forensic autopsies and instead view MRI as a supplementary examination. Autopsy and CT are considered the gold standard in forensic investigations of craniocerebral gunshot wounds since these examination modalities usually provide information related to the most relevant forensic questions, while the diagnostic opportunities for MRI are frequently unknown. MRI is known to be superior to CT for the detection of soft tissue injuries, while CT is appreciated for the detection of osseous injuries. However, using dedicated MRI sequences can yield equally good visualization of bone damage as CT and allow differentiation of the entrance and exit wounds and the detection of characteristic osseous injuries in craniocerebral gunshot wounds [31]. On CT, the wound tract is usually identified by bone splintering and bullet fragments along the bullet’s path and blood in the wound tract. However, in cases of massive bone damage and soft tissue destruction together with a longer postmortem interval, identification of the wound tract can be challenging or infeasible on CT, while MRI can still provide information on the wound tract as observed in case 1 of the present study. Likewise, at autopsy, examinations of cerebral tissue in cases with a longer postmortem interval can also be challenging, while in situ assessments of the cerebral tissue and the detection of hemorrhages by MRI are feasible [27]. Postmortem MRI can delineate relevant soft tissue injuries that may be missed macroscopically at autopsy, such as shearing injuries [30, 32], and the MRI examination can be reassessed for a second opinion at any time afterwards. Furthermore, the detection and localization of tiny injuries and pathologies on postmortem MRI can facilitate more precise sampling for histology. According to the findings in the present study, MRI may provide rapid information on cavitation injuries, whereas preparation of a sample for histological examination requires time due to formalin fixation. Additionally, the entire brain can be examined on MRI, while tissue blocks must be cut for histological examinations [3, 33]. With regard to the extent of cavitation injuries, tissue fixed in formalin and embedded in paraffin has been suggested to shrink by approximately 10% [3]. Postmortem MRI, however, enables three-dimensional in situ documentation of the entire cerebral tissue in actual size. An assessment of the vitality of a gunshot wound, which is feasible by immunohistochemical examinations of cerebral tissue at the border of the wound tract [33], is not currently feasible on MRI. However, MRI technology provides a large range of specific imaging sequences and analytical techniques, and their potential value for specific medico-legal questions regarding decedents must be further researched.

Postmortem MRI in the research field of forensic medicine has only been applied in studies with a small number of cases. The literature lacks large-scale studies to substantiate the diagnostic value of postmortem MRI in forensic medicine; therefore, the actual practical value of postmortem MRI in forensic case processing remains elusive. High costs and reliance on skilled personnel are frequently mentioned limitations to the implementation of MRI in (forensic) pathology. Revised strategies are essential to overcome these limitations and may increase the use of postmortem imaging considering declining autopsy rates [34–36].

## Conclusions

From a scientific perspective, further research evaluating a large number of subjects using 7 T MRI would be desirable to assess the diagnostic benefit of 7 T MRI for various forensic questions. With regard to investigations of craniocerebral gunshot wounds, this study demonstrates that 7 T MRI provides noninvasively acquired detailed information on soft tissue injuries with high spatial resolution. Hence, 7 T MRI may become a valuable examination tool for radiologic wound ballistics in the future of forensic science.

### Key points

1. MRI is valuable for radiologic wound ballistics.

2. Compared to 3 T MRI, 7 T MRI can provide more detailed visualization of brain and skull anatomy.

3. Compared to 3 T MRI, 7 T MRI can offer more sensitive detection of craniocerebral soft tissue injuries.

4. 7 T MRI shows the extent of temporary cavities (cavitation injuries) via dark/hypo intense regions adjacent to the wound tract.

5. 7 T MRI delineates microhemorrhages (microbleeds) with very high spatial resolution.
